# Transcriptional Regulation of PES1 Expression by c-Jun in Colon Cancer

**DOI:** 10.1371/journal.pone.0042253

**Published:** 2012-07-30

**Authors:** Wei Xie, Qin Feng, Yahui Su, Bin Dong, Jian Wu, Lin Meng, Like Qu, Chengchao Shou

**Affiliations:** 1 Key Laboratory of Carcinogenesis and Translational Research (Ministry of Education), Department of Biochemistry and Molecular Biology, Peking University Cancer Hospital & Institute, Beijing, China; 2 Key Laboratory of Carcinogenesis and Translational Research (Ministry of Education), Department of Pathology, Peking University Cancer Hospital & Institute, Beijing, China; University of Bari & Consorzio Mario Negri Sud, Italy

## Abstract

Pescadillo is a nucleolar protein that has been suggested to be involved in embryonic development and ribosome biogenesis. Deregulated expression of human pescadillo (PES1) was described in some tumors, but its precise roles in tumorigenesis remains unclear. In this study, we generated three monoclonal antibodies recognizing PES1 with high specificity and sensitivity, with which PES1 expression in human colon cancer was analyzed immunohistochemically. Out of 265 colon cancer tissues, 89 (33.6%) showed positive PES1 expression, which was significantly higher than in non-cancerous tissues (P<0.001). Silencing of PES1 in colon cancer cells resulted in decreased proliferation, reduced growth of xenografts, and cell cycle arrest in G1 phase, indicating PES1 functions as an oncogene. We then explored the mechanism by which PES1 expression is controlled in human colon cancers and demonstrated that c-Jun, but not JunB, JunD, c-Fos, or mutant c-Jun, positively regulated PES1 promoter transcription activity. In addition, we mapped −274/−264 region of PES1 promoter as the c-Jun binding sequence, which was validated by chromatin immunoprecipitation and electrophoretic mobility shift assays. Moreover, we demonstrated a positive correlation between c-Jun and PES1 expression in colon cancer cells and colon cancer tissues. Upstream of c-Jun, it was revealed that c-Jun NH2-terminal kinases (JNK) is essential for controlling PES1 expression. Our study, in the first place, uncovers the oncogenic role of PES1 in colon cancer and elucidates the molecular mechanism directing PES1 expression.

## Introduction

*Pescadillo* encodes a nucleolar protein with several motifs, including a BRCA1 C-terminal (BRCT) domain, clusters of acidic amino acids domains, several nuclear localization signals, and a conserved site for SUMOylation [1]. It was initially identified as a gene essential for zebrafish embryonic development [2]. The subsequent studies found that pescadillo was highly conserved from yeast to human [1], [3]–[5]. Human ortholog of Pescadillo (PES1) forms a stable complex with Bop1 and WDR12 (PeBoW complex), which is crucial for nucleolar localization and its function in rRNA processing [6]–[8]. BRCT-deleted or -mutated form of PES1 is less stable and can not be incorporated into the PeBoW complex [9]. Another nucleolar protein B23 physically interacts with PES1 and is involved in controlling the nucleolar localization of PES1 [10]. Pescadillo has been shown to play important roles in normal embryonic development, ribosome biogenesis, DNA replication, chromosomal stability, and cell cycle progression. Disruption of pescadillo or its orthologs in yeast, zebrefish, and mouse impaired embryonic development [2], [3], [11], [12]. PES1 plays a critical role in pre-rRNA processing and 60S ribosomal subunit maturation, through formation of the PeBoW complex [3], [7], [9], [13]. Besides, knockdown of PES1 induced cell-cycle arrest and decreased phosphorylation of retinoblastoma protein (Rb) [14]. Moreover, PES1 has been demonstrated to bind DNA directly and to regulate gene transcription [15], suggesting that PES1 is a multifunctional protein contributing to diverse biological processes.

Recently, deregulated expression of PES1 was found to be associated with cancer development [1], [16]–[18]. PES1 was abnormally upregulated in adult human glioblastomas [1], head and neck squamous cell carcinomas (HNSCCs) [19], and gastric cancer [20]. PES1 expression was also significantly increased in breast cancer cells and tissues at both mRNA and protein levels [21], and was possibly controlled by estrogen [22]. In addition, PES1 has been linked to the chromosomal instability [18], [23] and transformation of mammalian cells [16]. Despite of these findings, little is known about the precise role of PES1 in tumorigenesis and the factors directing PES1 expression remain to be determined.

In the present study, we demonstrated high expression of PES1 in colon cancer tissues. We found that PES1 plays an oncogenic role in promoting proliferation of colon cancer cells and tumor formation in the nude mice model. Transcriptional factor c-Jun enhances PES1 expression by binding to the promoter region of PES1 and positive correlation between c-Jun and PES1 expression is evident in colon cancer cells and tissues.

## Materials and Methods

### Ethics Statement

The collection of tissue samples was approved and supervised by the Research Ethics Committee of Peking University Cancer Hospital & Institute. Written Informed Consents were obtained from all patients prior to operation. Animal studies, including antibody generation and xenograft tumor model, were approved and supervised by Research Ethics Committee of Peking University Cancer Hospital & Institute.

### Materials

Expression plasmids for c-Jun, JunB, JunD and c-Fos were kindly provided by Dr. Zhihua Liu (Peking Union Medical College, China). The double mutant c-Jun-S63A/S73A was a gift from Dr. Dirk Bohmann (University of Rochester Medical Center). pGL3-Basic and pRL-SV40 plasmids were purchased from Promega (Madison, WI, USA). Antibodies against c-Jun (H-79, sc-1694) and c-Fos (sc-52) were from Santa Cruz Biotechnology (Santa Cruz, CA, USA). Antibodies against JunB (D253, BS1196) and JunD (V249, BS1198) were from Bioworld Technology (St. Louis Park, MN, USA). Antibody against JNK1 (ab27709) was from Abcam (Cambridge, MA, USA). Anti-β-actin was purchased from California Bioscience (Coachella, CA, USA). Anti-GAPDH was from ProteinTech (Chicago, IL, USA). Kinase inhibitors U0126 and LY294002 were purchased from Cell Signaling (Danvers, MA, USA). SP600125 was purchased from Sigma (Sigma-Aldrich, St. Louis, MO, USA).

### Generation of monoclonal antibody

Hybridomas secreting anti-PES1 antibodies were generated according to a standard protocol. Briefly, five female BALB/c mice (purchased from Animal Center of the Chinese Academy of Medical Sciences) were immunized with recombinant GST-PES1 protein (50 µg per mouse) emulsified in Freund's adjuvant (Sigma) four times at 3-week intervals. On day 4 after the final immunization, the spleen was removed from one immunized mice and the cells were fused with Sp2/0 myeloma cells, using 50% (v/v) polyethylene glycol 4000 (Merck, Darmstadt, Germany). Hybridomas were cultured in HAT (hypoxanthine, aminopterine, and thymidine, Sigma) selection medium. After 14 days, the supernatants were harvested and screened for the presence of specific anti-PES1 monoclonal antibodies (mAbs) by ELISA. The stable hybridomas were expanded and the mAbs were purified by protein A/G-coupled Sepharose beads (Invitrogen) chromatography.

### Cell culture and transfection

HCT116, SW480, RKO, and AGS cells were obtained from American type culture collection (ATCC). Cells were maintained in DMEM or RPMI-1640 medium (Hyclone, Logan, UT, USA) supplemented with 10% fetal bovine serum at 37°C in a 5% CO_2_ environment. For transfection, cells were seeded in culture plates, grown to 50–80% confluency and transfected with plasmids or siRNA using Lipofectamine 2000 (Invitrogen, Carlsbad, CA, USA) according to the manufacturer's protocol. In DNA plasmid transfection, 1.6 µg DNA was used per well for 12-well culture plate. In siRNA transfection, 50 pmol siRNA was used per well for 12-well culture plate. Following transfection, cells were incubated for another 48–72 hr before being harvested for the luciferase assay or gene expression testing. Alternatively, after transfection for 48 hr, cells were treated with the indicated kinase inhibitors for another 36 hr. siRNA sequences used to knock down the expression of c-Jun and JNK1 were as follows: siRNA-c-Jun, GCAAAGAUGGAAACGACCUUCUAUGTT; siRNA-JNK, AAAGAAUGUCCUACCUUCUTT.

### Western blot analysis

Cells were lysed in a modified RIPA buffer containing 50 mM Tris, pH 7.5, 150 mM NaCl, 1 mM EDTA, 1 mM Na_3_VO4, 20 mM NaF, 1% NP-40, 0.5% sodium deoxycholate, 0.1% SDS and 1× protease inhibitor cocktail (Roche, Mannhelm, Germany). Proteins were separated by SDS-PAGE and transferred to nitrocellulose membranes. After blocking with 5% fat-free milk in phosphate-buffered saline (PBS) for 2 hr at room temperature, the membranes were incubated with the primary antibody overnight at 4°C followed by incubation with secondary antibody conjugated to horseradish peroxidase (Jackson, West Grove, PA). Protein bands were visualized using enhanced chemiluminescence detection (Pierce, Rockford, IL, USA). Relative optical densities of protein bands to that of loading control (GAPDH) were quantified by Scion Image software.

### Immunohistochemical analysis

For immunohistochemical staining, all clinical samples were fixed in freshly prepared 10% neutral buffered formalin, embedded in paraffin, and cut into 5 µm sections. After baking at 60°C overnight, sections were dewaxed and rehydrated. Thereafter, antigen retrievaling was carried out via high pressure cooking in EDTA (pH 8.0, Zymed). Endogenous peroxidase activity was blocked by incubation in 3% hydrogen peroxide for 10 min at room temperature. After blocking with 5% fat-free milk, sections were incubated with specific PES1 mAb 3B1 (1∶500) at 4°C overnight followed by incubation with secondary antibody from the EnvisionTM kit (Dako Cytomation, Cambridge, UK) for 45 min at room temperature. The reaction product was visualized with diaminobenzidine (DAB, Sigma) for 5 min at room temperature and the sections were counterstained with hematoxylin. Purified IgG from normal mouse serum was used as a negative control. The results were evaluated independently by two pathologists (Q.F. and B.D.). The specimen with more than 20% immunostaining cells was classified as the positive case.

### Inhibition of PES1 by RNA interference

To stably knock down endogenous PES1 expression, we used lentivirus packing shRNA expression vector (purchased from GenePharma, Shanghai, China) to infect cells. Target cells were infected with lentivirus for 24–48 hr according to manufacturer's instruction. The RNAi oligonucleotides sequence used to knock down endogenous PES1 expression is as follows: PES1-RNAi-1, GGAACACTGTAGAGCGTTTAA; and PES1-RNAi-2, GAAGATGCAGAGGCTGGTTCA.

### Proliferation assay

Cells were seeded on 96-well plates at initial density of 1.0×10^3^ per well. At each time point, cells were stained with sterile MTT (Methylthiazolyldiphenyl-tetrazolium, 0.5 mg/ml, Sigma) for 4 hr at 37°C, followed by removal of the culture medium and addition of 150 µl dimethyl sulfoxide (DMSO). The absorbance was measured using a microplate reader at a wave-length of 490 nm. All experiments were performed in triplicate.

### Colony formation assays

Cells were plated on 6-well plates (1×10^3^ cells per well) and cultured for two weeks. The colonies were stained with 0.5% crystal violet for 30 min after fixation with methanol for 30 min at room temperature.

### Cell cycle analysis

Cells were harvested by centrifugation, washed twice with ice-cold PBS and fixed in 75% ethanol (in PBS) at 4°C overnight. After washing twice with cold PBS, cells were re-suspended in PBS containing 0.1 mg/ml RNase (Sigma). After 30 min at 37°C, the cells were re-suspended in PBS containing 50 µg/ml propidium iodide (PI) and then analyzed with a flow cytometer (BD, CALIBUR). The cell cycle distribution was calculated using Cell Quest and Mod-fit software.

### Tumor xenograft assay

nu/nu female mice (from Vital River Laboratories, Beijing, China) between 7 and 8 weeks were used for *in vivo* studies. Each experimental group consisted of 4–5 nude mice. 4×10^6^ cells in 100 µl PBS were subcutaneously injected into the armpit of nude mice. After 22 days, mice were scarified and tumors were removed and weighted. The data shown are means ± SD of mice in each group.

### Gene expression microarray

RNA was extracted from cells with Trizol reagent (Invitrogen) according to the protocol suggested by provider. Gene expression profiles in PES1-silenced HCT116 and control cells were examined using Agilent Human Genome CGH Microarray 44K. After normalization, the fold change expression was calculated. A P value less than 0.05 and the fold-change threshold 2 were chosen to identify the statistically significant transcriptional alterations.

### Luciferase assay

To analyze the promoter activity of PES1, 5′-flanking region plus 172 bp of transcribed PES1 sequence was generated by PCR using the following primers: Forward, 5′- CCGCTCGAGCTGGCATTATCCTGGAGTCAC-3′ (XhoI site underlined), Reverse, 5′- CCCAAGCTTGAAAAGAGTCGACCCCATGC-3′ (HindIII site underlined). The amplified fragment from the genomic DNA of HCT116 cells was inserted into pGL3-basic plasmid vector, and the resulting plasmid was named as pGLB-PES1(−2060/+172). The luciferase reporter plasmids, pGLB-PES1(−1413/+172), pGLB-PES1(−902/+172), pGLBPES11(−416/+172), pGLB-PES1(−315/+172), pGLB-PES1(−282/+172), pGLB-PES1(−274/+172), pGLB-PES1(−264/+172), pGLB-PES1(−254/+172) and pGLB-PES1(−75/+172) were generated from pGLB-PES1(−2060/+172). pGLB-PES1-promoter or 5′-deletion constructs were cotransfected with pRL-SV40 into cells. After transfection for 72 hr, cells were harvested in Passive Lysis Buffer (Promega) and the cell lysates was analyzed for luciferase activity with the Dual Luciferase Reporter Assay System (Promega) following the manufacturer's instructions. Luciferase activity was normalized to Renilla luciferase activity.

### Chromatin immunoprecipitation (ChIP)

Cells were fixed with 1% formaldehyde for 10 min at 37°C, followed by washing twice with ice-cold PBS and harvested by scraping. After centrifugation, cell pellets were lysed in 400 µl of lysis buffer (50 mM Tris-HCl, pH 8.1, 1% SDS, 5 mM EDTA, 1 mM PMSF, 1× protease inhibitors cocktail). Samples were incubated on ice for 10 min and sonicated four times for 15 seconds each time at intervals of 15 seconds to obtain chromatin fragments of about 200–1000 bp nucleotides. Samples were centrifuged at 12,000 rpm, 4°C for 10 min. After removal of a control aliquot (20 µl of supernatants was diluted in 80 µl of dilution buffer and stored at −20°C), supernatants were diluted with 9 volumes of ChIP dilution buffer (20 mM Tris-HCl, pH 8.0, 150 mM NaCl, 2 mM EDTA, 1% NP-40, 1 mM PMSF, 1× protease inhibitors cocktail). Samples were preincubated with protein A/G beads plus 10 µg salmon sperm DNA at 4°C for 2 hr, and then centrifuged at 1000 rpm, 4°C for 2 min. Supernatants were incubated at 4°C overnight with 0.2 µg c-Jun-specific antibody or IgG that had been preincubated with protein A/G beads and 10 µg salmon sperm DNA. The beads were then washed with TSE I (0.1% SDS, 2 mM EDTA, 20 mM Tris-HCl, pH 8.0, 150 mM NaCl, 1% NP-40), TSE II (0.1% SDS, 1% NP-40, 2 mM EDTA, 20 mM Tris-HCl, pH 8.0, 500 mM NaCl),TSE III (10 mM Tris-HCl, pH 8.0, 250 mM LiCl, 1 mM EDTA, 1% NP-40, 1% deoxycholate) buffers in turn for one time, followed by washing twice with TE buffer (pH 8.0). After the final washing, the immunoprecipitates were eluted and reverse cross-linked by incubation overnight at 65°C in elution buffer (1% SDS, 100 mM NaHCO_3_). DNA was then purified with a PCR purification kit (Qiagen, Hilden, Germany). Eluted DNA was PCR-amplified with primers encompassing the c-Jun binding site of PES1 promoter. The chromosomal DNA input and ChIP DNA with nonspecific IgG were subjected to the same PCR amplification. PCR products were separated on a 2% agarose gel containing ethidium bromide and detected via ultraviolet illumination. The following primers were used for PCR. Specific primers for c-Jun binding (Primer-S, 139 bp fragment, −363 to −225 region of PES1 promoter), Forward primer, CTTGACAACGCAATCCTATCG; Reverse primer, CCTGATGACGATTCATTGACTGT; and negative control primers (Primer-N, 110 bp fragment, −1473 to −1364 region of PES1 promoter), Forward primer, CAACTAGCTGGGGTTACAGG; Reverse primer, GAGATCAGGAGTTTGAGAC. The following antibodies were used: rabbit polyclonal anti-c-Jun (H-79, Santa Cruz) and rabbit normal IgG (Santa cruz).

### Electrophoretic mobility shift assay (EMSA)

Cells were washed twice with ice-cold PBS and harvested by scraping. After centrifugation, cell pellets were resuspended in 160 µl ice-cold buffer A (10 mM Hepes, 10 mM KCl, 0.1 mM EDTA, 1 mM DTT, 0.5 mM PMSF) and incubated on ice for 20 min. And then another 40 µl buffer A containing 2.5% NP-40 was added to the sample with intermittent vortexing for 10 s. After centrifugation 12,000 rpm at 4°C for 5 min, cell pellets were resuspended in 40 µl ice-cold buffer B (20 mM Hepes, 400 mM KCl, 1 mM EDTA, 1 mM DTT, 1 mM PMSF) and placed on ice with intermittent vortexing for 25 min. Cell debris was removed by centrifugation. Supernatants containing nuclear protein were stored at −70°C. Binding assays were performed by incubating 8 µg of nuclear protein in the binding buffer (10 mM Tris-HCl, pH 7.5, 50 mM KCl, 1 mM DTT, 10 µg salmon sperm DNA) with 30 fmol biotin-labeled oligonucleotides in a final volume of 20 µl for 20 min at room temperature. For cold competition, a 1000 fold excess of the unlabeled oligonucleotides was added to the binding reaction 20 min prior to addition of the labeled oligonucleotides. For supershift assays, 0.1 µg antibody was incubated with binding mixtures containing 10 fmol biotin-labeled probe for 40 min at room temperature. DNA-protein complexes were separated by electrophoresis through a 6% native polyacrylamide gel in 0.5× TBE at 100 V for 180 min at 4°C. The gels were then transferred to a positively charged nylon membrane. The bound probes were visualized by HRP-conjugated Streptavidin and chemiluminescent substrate (Thermo Chemiluminescent nucleic acid detection kit). The oligonucleotide probes used in EMSA were as follows: Probe1, CTTCCGCCCCTCTCCGTCCCAACATGCAAC (−284/−255 sequence of PES1 promoter); Probe-W (containing previously identified wild-type c-Jun binding sequences), GCCCGCAGTGCTGGGCGGGGCGCTGACTCACCCGGGCCCGGG; Probe-Am (containing mutated AP-1 binding sequences), GCCCGCAGTGCTGGGCGGGGCGCGTCGGATCCCGGGCCCGGG [24]. The probes were synthesized and labeled with biotin by SBS Genetech (Beijing, China). The complementary oligonucleotides were annealed in 20 mM Tris (pH 7.6), 50 mM NaCl, 10 mM MgCl_2_, and 1 mM DTT.

### Real-time reverse transcription polymerase chain reaction

Total cellular RNA was extracted from cells with TRIzol reagent (Invitrogen) and reverse transcribed to cDNA using ImProm-II™ Reverse Transcription System (Promega). The quantitative PCR was carried out with the ABI StepOne Real-Time PCR system (Applied Biosystems) using SYBR Green Realtime PCR Master Mix (TOYOBO, Osaka, Japan) according to the manufacturer's instructions. All reactions were carried out in triplicate. Relative gene expression was calculated using the 2^−ΔΔCt^ method following the manufacturer's instructions. Housekeeping gene glyceraldehyde-3-phosphate dehydrogenase *(gapdh)* was used as internal controls to normalize *PES1* mRNA expression. The following primers were used: *PES1*, forward CCCAAGCAGAGGCAAAGG, reverse CTGACATCTCCCCATCGG; *c-Jun*, forward CGCCCCTGTCCCCCATCG, reverse TGTGCCACCTGTTCCCTG; *gapdh*, forward CATCAAGAAGGTGGTGAAGCAG, reverse CGTCAAAGGTGGAGGAGTGG. Primers of quantitative RT-PCR for validating the results of Microarray were listed in Table S1.

### Statistical analysis

Data analysis was performed using SPSS 13.0 (SPSS, Inc., Chicago, IL). A two-tailed independent-sample *t* test was used to determine the significance of differences between different experimental groups. The correlation of c-Jun and PES1 in colon cancer tissues was analyzed by Pearson correlation coefficient with SPSS software. Differences were considered statistically significant at P<0.05.

## Results

### PES1 is overexpressed in colon cancer

We firstly generated three monoclonal antibodies (mAbs) recognizing human PES1. Through ELISA and Western blot analysis, these three mAbs were shown to specifically bind to GST-PES1 protein, but not to GST (Fig. S1A and S1B). Furthermore, these mAbs could recognize endogenous PES1 in Western blot assay (Fig. S1C). Next, we confirmed the specificity of mAb 3B1 by analyzing total cell lysates of control and PES1 shRNA-transfected AGS gastric cancer cells. A protein band corresponding to the expected molecular size of PES1 (69 kD) was ablated by a short hairpin RNA (shRNA) against PES1 (Fig. S1D). In addition, we demonstrated that mAb 3B1 could be utilized in ELISA, Western blot and immunocytochemistry analysis with higher sensitivity and affinity, in comparison to a commercial antibody (Fig. S1E–G).

**Figure 1:**
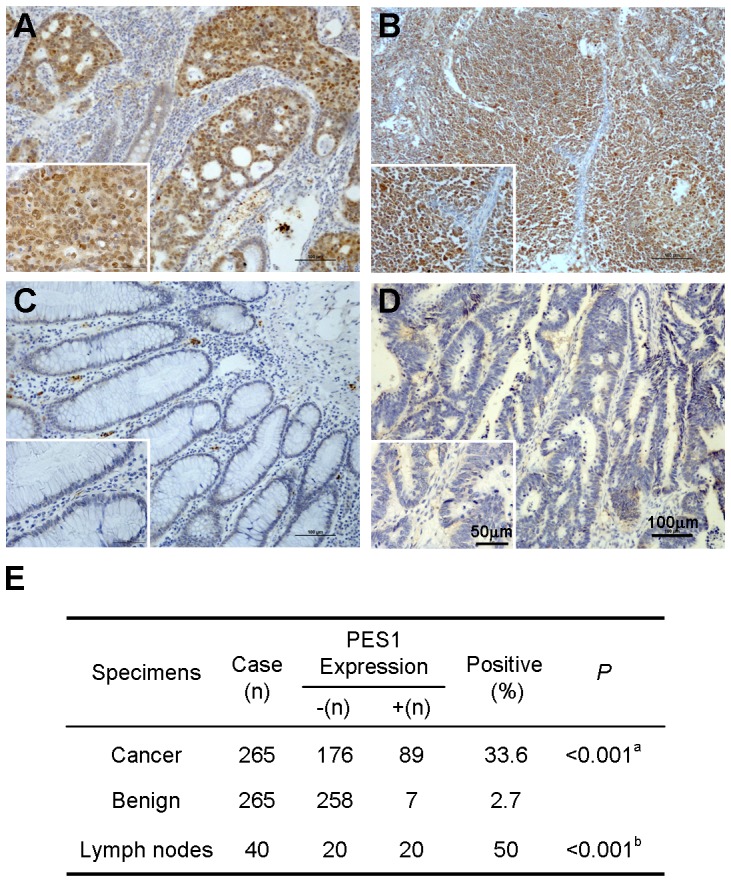
PES1 is overexpressed in colon cancer. Immunohistochemical analysis of PES1 expression in human colon cancer tissues. The figures show the strongly nuclei staining of PES1 in colon cancer tissues (A) and lymph nodes (B). Negative staining of PES1 in non-cancerous tissues adjacent to tumor was shown in (C). Normal mouse IgG was used as a negative control in colon cancer tissues and shown in (D). Representative low (×100) and high (×400) magnification are shown. (E) Summary of PES1 expression in human colon cancer tissues, matched adjacent tissues, and lymph nodes. a indicates significant difference between colon cancer versus matched adjacent tissue. b indicates significant difference between lymph nodes versus adjacent noncancerous tissues.

To examine the expression of PES1 in colon cancer tissues, we performed immunohistochemistical analysis of human colon cancer tissues and matched adjacent tissues with mAb 3B1. As shown in Figure 1A and 1B, PES1 exhibited strong nuclear staining (brown nuclei) in cancer cells and lymph nodes, respectively. Out of the 265 colon cancer tissues, 89 (33.6%) were positive for PES1 expression, whereas only 2.7% (7/265) of adjacent tissues showed PES1 staining (Fig. 1E). The difference of PES1 expression between the colon cancer tissues and non-cancerous tissues was significant (P<0.001). Furthermore, 20/40 (50%) of lymph nodes showed positive PES1 staining, also significantly higher (P<0.001) than in non-cancerous tissues. Therefore, these results indicated PES1 expression was up-regulated in human colon cancer tissues.

### PES1 promotes colon cancer cell proliferation and growth *in vitro* and *in vivo*

**Figure 2:**
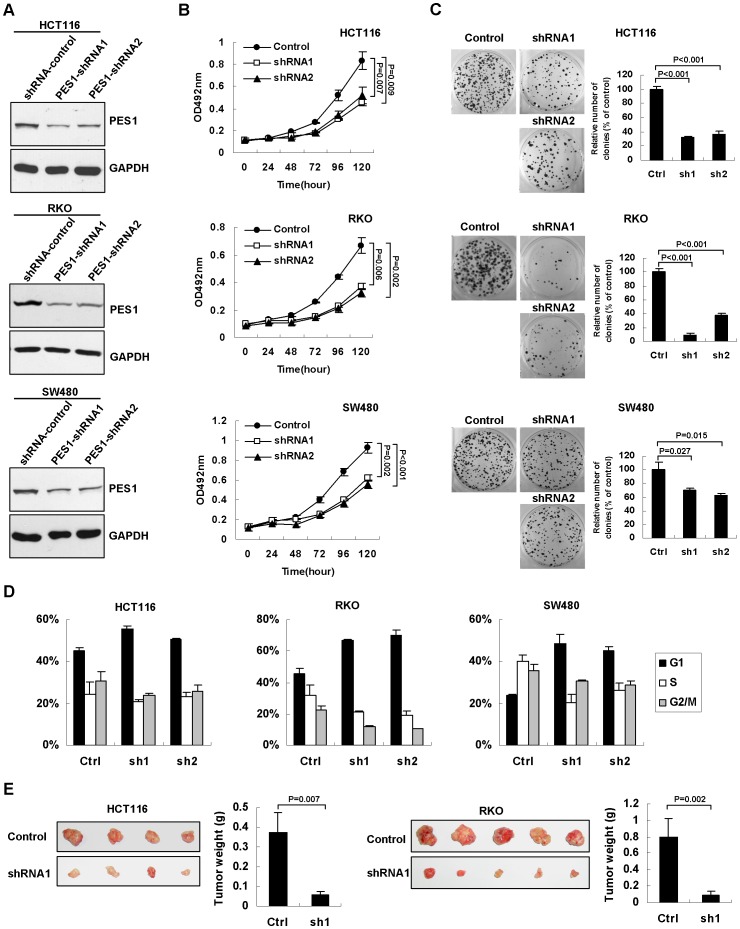
PES1 promotes colon cancer cells proliferation and growth of xenografts. (A) Western blot analysis for PES1 protein in HCT116, RKO, and SW480 cells transduced with shRNA-control and PES1 shRNA constructs (PES1-shRNA-1 and PES1-shRNA-2), respectively. (B) Growth curves of indicated cells transduced with PES1 shRNAs, as examined by MTT assay. Values presented represent means ± SD from three independent experiments. (C) Silencing of PES1 decreased colony formation. The photos demonstrate results of colony-formation assay of cells in the plate (left panel). The relative colony numbers (right panel) were obtained from three independent experiments. (D) Silencing of PES1 resulted in cell cycle arrest. Quantification of cell cycle distributions was derived from three independent experiments. Values represent means ± SD. (E) Silencing endogenous PES1 inhibits tumor growth of HCT116 and RKO cell in nude mice. The right panel shows the tumor weight of shRNA-control and shRNA-1. Values presented represent means ± SD.

To investigate the biological function of PES1 in the pathogenesis of colon cancer, lentiviral-mediated stable ablation of PES1 was performed in HCT116, RKO, and SW480 colon cancer cells with two pairs of shRNAs. Both shRNA effectively silenced the expression of endogenous PES1 in these cell lines (Fig. 2A). In vitro assays uncovered that depletion of endogenous PES1 resulted in significant inhibitions of cell proliferation (Fig. 2B) and colony formation (Fig. 2C). Cell cycle analysis further demonstrated that cells tended to be accumulated in G1 phase, but less in S phase, upon PES1 ablation (Fig. 2D), indicative of G1/S arrest. However, no significant difference was found in the steady levels of apoptotic cells between control shRNA and PES1-specific shRNAs-transfected cells (data not shown). Furthermore, we inoculated the PES1-ablated HCT116 and RKO cells into nude mice to examine the effect of PES1 on xenograft tumor formation. As shown in Fig. 2E, silencing of PES1 inhibited tumor growth *in vivo*.

To gain insight into PES1's biological functions, we performed microarray analysis with PES1 stably silenced HCT116 cells and control cells. Genes with same pattern of changes in both shRNA-1- and shRNA-2-transfected cells were picked up. A total of 633 genes were identified to be differently expressed (either 2 fold increase or decrease) upon PES1 silencing, including 305 up-regulated and 328 down-regulated genes. Bioinformatical analysis was carried out to identify pathways affected by PES1 ablation with Gene Ontology Analysis tool (Fig. S2A). These pathways were ranked according to the significance (P values). Consistent with the results of functional analysis, genes related to control of cell proliferation constitute the primary category affected by PES1 ablation (Fig. S2A). We then examined expression of four down-regulated genes (*bcl2*, *fgf9*, *satb1*, and *avp*) and four up-regulated genes (*id3*, *msx2*, *gdf11*, and *smad3*) by quantitative RT-PCR in HCT116 and SW480 cells, and confirmed the results of microarray analysis (Fig. S2B).

### PES1 expression is regulated by c-Jun in colon cancer cells

**Figure 3:**
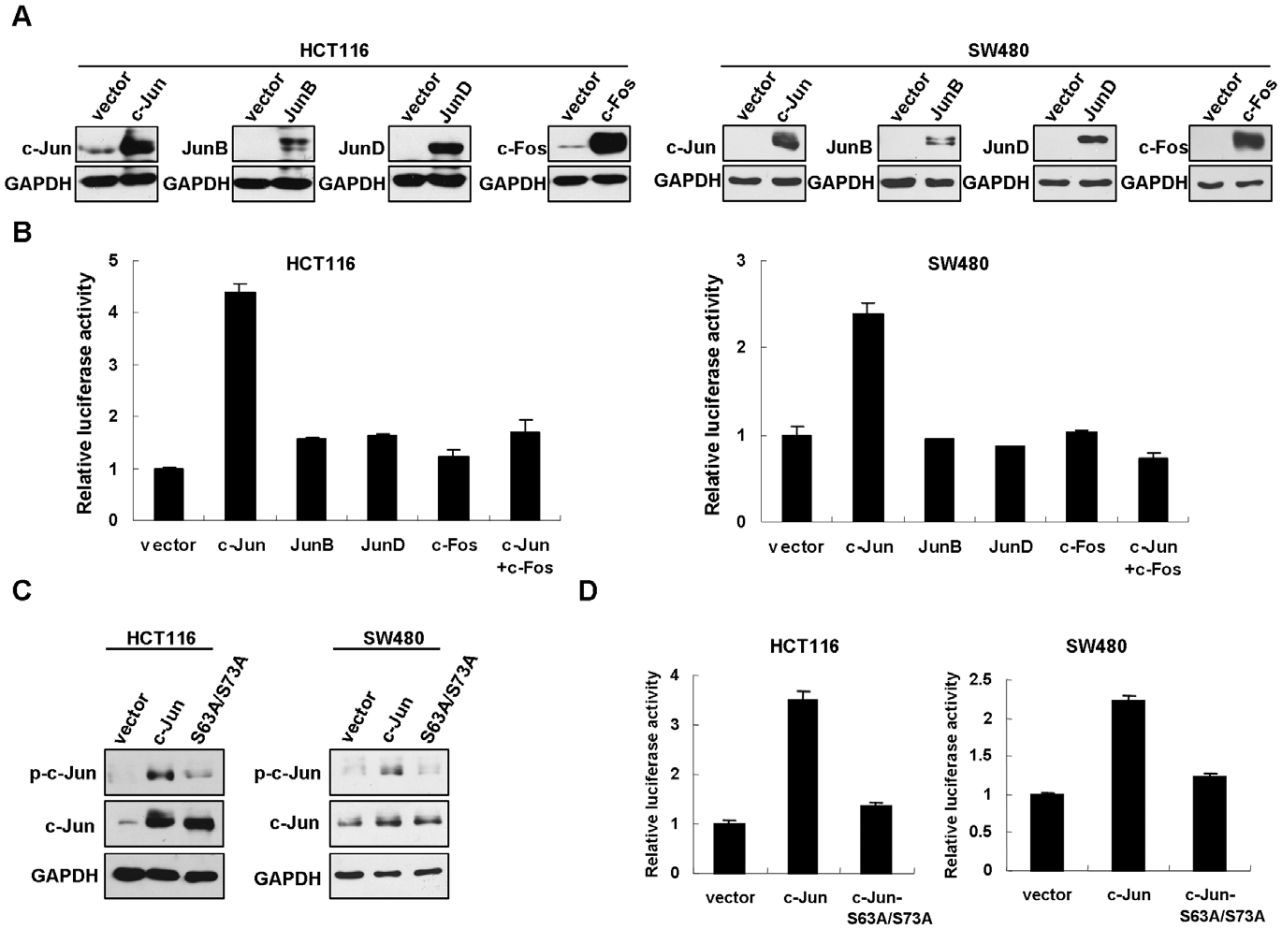
PES1 promoter activity is upregulated by c-Jun. (A) Western blot analysis detecting the ectopic c-Jun, JunB, JunD and c-Fos in HCT116 and SW480 cells. GAPDH is shown as a loading control. (B) Luciferase reporter assay in colon cancer cells. pGLB-PES1-promoter (−2060/+172) was co-transfected with indicated plasmids into HCT116and SW480 cells. Relative luciferase activity was normalized to Renilla luciferase activity. The empty vector was used as control and the relative luciferase activity was set to 1. (C) Expression of wild type and c-Jun-S63A/S73A in HCT116 and SW480 cells. Phosphorylation of c-Jun was also detected. (D) pGLB-PES1-promoter (−2060/+172) was co-transfected with c-Jun or c-Jun-S63A/S73A into HCT116 and SW480 cells, then the reporter assay was performed as in (B).

We were interested to find out the factor(s) controlling PES1 overexpression in colon cancer tissues. Preliminary data suggested that there were higher level of PES1 mRNA expression in colon cancer tissues than in match adjacent tissues, but no deregulated methylation was found in the promoter region of PES1 gene (data not shown). Moreover, multiple potential transcription factor binding sites within PES1 promoter were identified, including activator protein-1 (AP-1) binding sites. To better understand the transcriptional regulation of PES1, 2060 bp of the 5′-flanking sequence of PES1 gene and 172 bp of the transcribed sequence were cloned from HCT116 cells and subcloned into pGLB luciferase reporter plasmid. We next investigated whether AP-1 could regulate PES1 transcription. The transcription factor AP-1 consists of several components, such as c-Jun, JunB, JunD and c-Fos, which form homo- or hetero-dimer to bind the promoter sequences of downstream genes [25]. The regulation of these AP-1 subunits on PES1 promoter was examined by luciferase reporter assay. The pGLB-PES1-promoter (−2060/+172) was co-transfected with c-Jun, JunB, JunD or c-Fos into HCT116 and SW480 cells, whose expression was validated by Western blot (Fig. 3A). PES1 promoter activity was greatly increased by c-Jun, but minimally by other subunits (Fig. 3B). We also tested synergistic effects of c-Jun and c-Fos on PES1 promoter activity. To this end, pGLB-PES1-promoter (−2060/+172) was co-transfected with c-Jun plus c-Fos. The luciferase activity induced by co-transfecting with c-Jun plus c-Fos was not significantly increased and even lower than that by c-Jun alone (Fig. 3B), suggesting that heterodimerization between c-Jun and c-Fos is inadequate to foster the promoter activity of PES1. Phosphorylation of Serine-63 and Serine-73 in the NH_2_-terminal transactivation domain of c-Jun by JNK (c-Jun NH_2_-terminal kinases) is essential for the transcriptional activity of c-Jun [26]. We noticed that substitution of these serine residues with alanines (c-Jun-S63A/S73A) greatly impaired the phosphorylation (Fig. 3C) and PES1 promoter activity (Fig. 3D), suggesting that phosphorylation of c-Jun is critical for activating PES1 expression.

### c-Jun regulate PES1 promoter activity by directly binding to PES1 promoter

**Figure 4:**
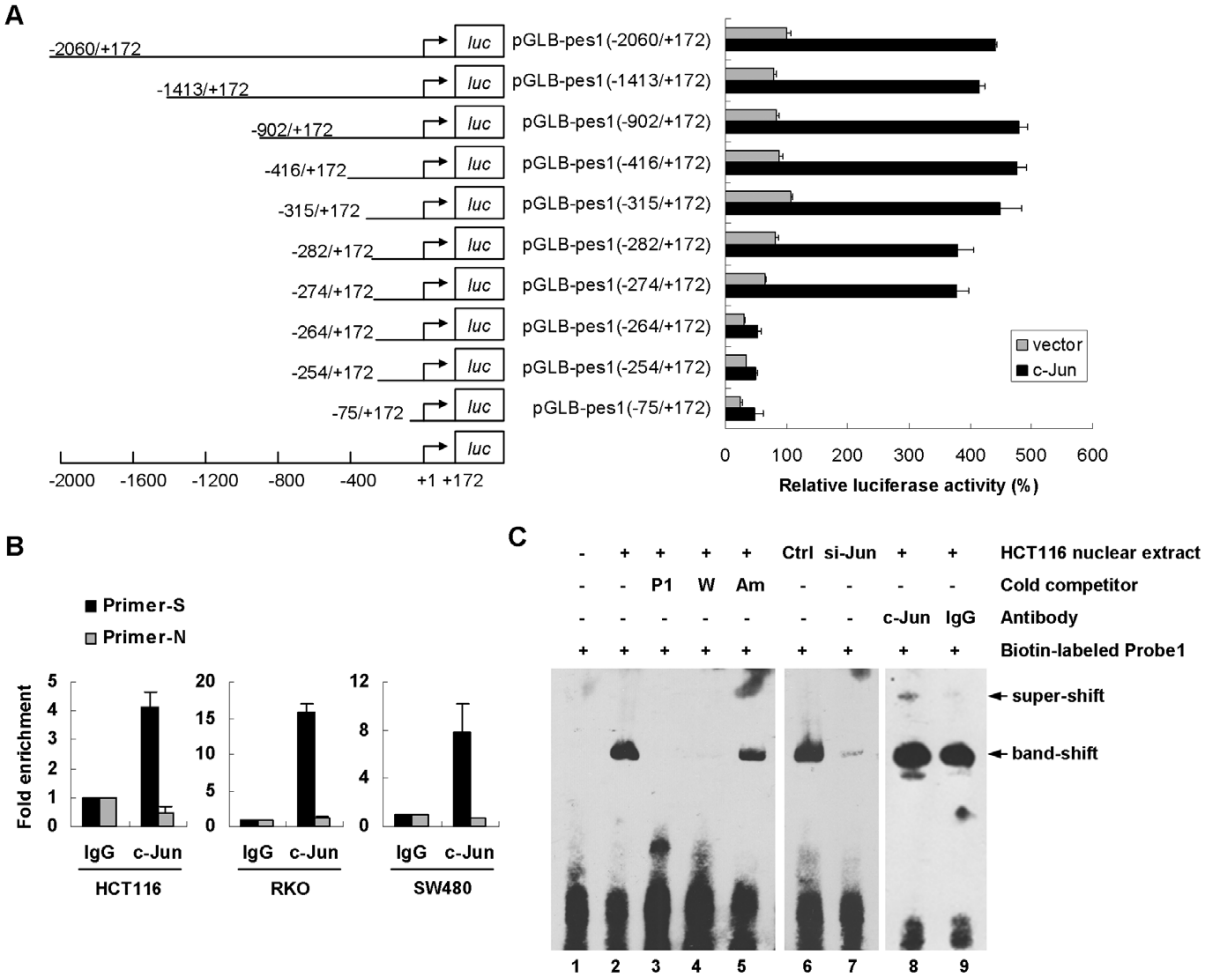
Direct binding of c-Jun to PES1 promoter. (A) Mapping of the binding region of c-Jun on PES1 promoter by 5′-deletion analysis. Schematic representation of the PES1 promoter 5′-deletion constructs used for luciferase repoter assay is shown on the left. 5′-Deletion constructs were cotransfected with c-Jun or control vector into HCT116 cells. Luciferase activity (right) was normalized to Renilla luciferase activity and then shown relative to that of HCT116 cells transfected with pGLB-PES1-promoter (−2060/+172), which was set to 100%. (B) Association of c-Jun with the PES1 promoter in cells was detected using the qChIP assay. Cross-linked chromatin isolated from HCT116, RKO, and SW480 cells was immunoprecipitated with anti-c-Jun and IgG control. The associated chromosomal DNA fragments were amplified with specific primers (Primer-S, 139 bp fragment) and negative control primers (Primer-N, 110 bp fragment). Quantitative PCR was performed and the relative binding to IgG was set as 1. Values represent means ± SD. (C) Binding of c-Jun protein to the PES1 promoter using EMSA assay. Biotin-labeled Probe1 (−284/−255 sequence) was incubated without (lane 1) or with (lane 2–9) nuclear extract proteins prepared from HCT116 cells. Several cold competitor probe were used for competition reaction at 1000-fold molar excess, including unlabeled Probe1 (P1, lane3), unlabeled probe-W (containing identified wild-type c-Jun binding sites, lane 4) and unlabeled probe-Am (containing mutated AP-1 binding site, lane 5). Nuclear extract deleted for c-Jun was used (siRNA-control, lane 6, and siRNA-c-Jun, lane 7). Anti-c-Jun antibody was added to the binding reaction for super-shift assay (lane 8) and nonspecific IgG was used as control (lane 9).

To map the c-Jun binding site on the PES1 promoter, a series of 5′-deletion mutants were generated and analyzed by co-transfection with c-Jun. Compared with region −2060/+172, further deletion, *i.e.* −1413/+172, −902/+172, −416/+172, −315/+172, −282/+172, and −274/+172, did not markedly change the reporter activity, but further deletion from −274 to −264 caused an ∼80% reduction in luciferase activity (Fig. 4A). Meanwhile, we analyzed the deletion mutants in the absence of exogenous c-Jun, and also found that deletion from −274 to −264 markedly reduced the reporter activity (Fig. 4A).These data suggest −274/−264 is a potential c-Jun binding sequence on the PES1 promoter. We next carried out chromatin immunoprecipitation (ChIP) assay to substantiate c-Jun's binding to the PES1 promoter. With an anti-c-Jun antibody, immunoprecipitated chromosomal DNA was subjected to quantitative PCR using primers designed to amplify the PES1 promoter sequence harboring the −274/−264 region. Results showed that c-Jun indeed interacted with the PES1 promoter region in HCT116, RKO, and SW480 cells, but not with sequence amplified with non-specific primers (Fig. 4B). Next the electrophoretic mobility shift assay (EMSA) was performed. Nuclear extracts from HCT116 cell were incubated with biotin-labeled Probe1 (−284/−255 of PES1 promoter) containing the candidate c-Jun binding sequence (−274/−264). This binding reaction generated one complex which was labeled as band-shift (Fig. 4C, lane 2). To confirm the specificity of such binding, several cold competitor probes were used for competition reaction, including unlabeled Probe1 (P1), unlabeled probe-W (containing previously characterized wild-type c-Jun binding sequences) and unlabeled probe-Am (containing mutated AP-1 binding sequences) [24]. Both cold P1 and probe-W could inhibit the formation of band-shift (Fig. 4C, lane 3 and 4), however probe-Am had no obvious inhibition (lane 5). Besides, with nuclear extract from HCT116 cells transfected with c-Jun-specific siRNA, we found that the band-shift was weakened (lane 6 and 7). Once an antibody against c-Jun was co-incubated with the binding reaction, a high molecular weight band emerged (super-shift) (lane 8), but IgG had no such effect (lane 9). These results validated the specific interaction between c-Jun and the PES1 promoter region *in vitro*.

### Correlation between c-Jun and PES1 expression in colon cancer cells and tissues

**Figure 5:**
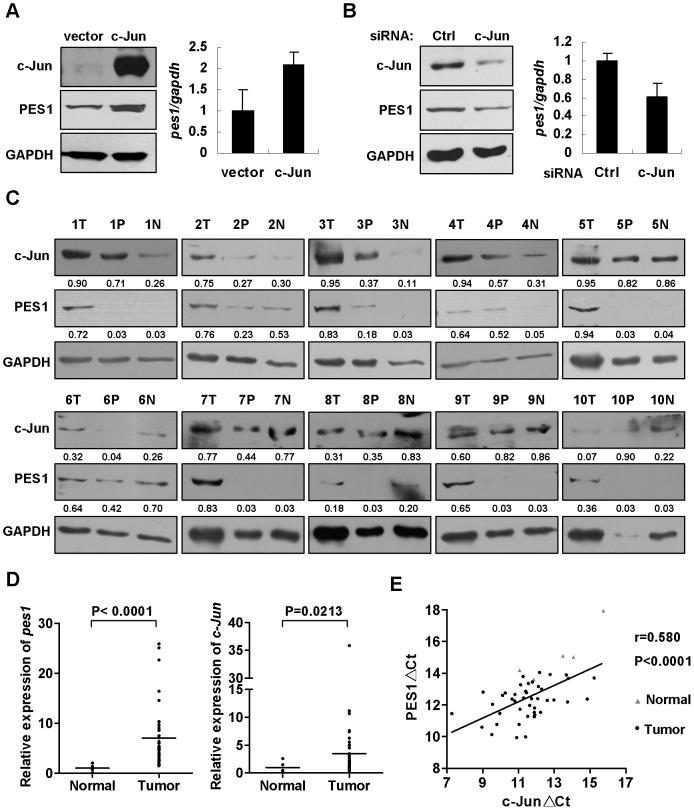
Correlation between c-Jun and PES1 expression in colon cancer cells and tissues. (A) In HCT116 cell, ectopic c-Jun upregulated endogenous PES1 at mRNA (quantitative RT-PCR, Right) and protein levels (Western blot, Left). (B) SW480 cells were transfected with 50 pmole c-Jun-specific or control siRNA for 72 hr. Expressions of c-Jun and PES1 were at protein (left) and mRNA (right) levels were examined. GAPDH is shown as a loading control in Western blot and *gapdh* is used as a housekeeping gene in quantitative RT-PCR. (C) Western blot analysis of PES1 and c-Jun expression levels in 10 sets of colon cancer tissues. T indicates colon cancer tissue; P, noncancerous tissue adjacent to tumor; N, distant noncancerous tissue from the surgical margin. Detection of GAPDH was used as loading control. Relative optical densities of PES1 and c-Jun to that of GAPDH were quantified by Scion Image software. (D) Gene expression levels of *PES1* and *c-Jun* were detected by quantitative real-time RT-PCR with ORIGENE TissueScan RT chip, normal = 5, tumor = 43. *Gapdh* was used as a housekeeping gene. A relative quantitative analysis was performed using ΔΔCt method (2^−ΔΔCt^). The horizontal lines show mean values. (E) PES1 and c-Jun gene expression have a correlation coefficient of r = 0.580, with a probability of P<0.0001. ▴ normal, • tumor.

Next we explored whether c-Jun could up-regulate PES1 transcription and protein expression in cells. As expected, ectopic c-Jun upregulated endogenous PES1 at mRNA and protein levels (Fig. 5A). Conversely, we noted that transient silencing of c-Jun diminished mRNA and protein level of PES1 (Fig. 5B). To support c-Jun-regulated PES1 expression in colon cancer cells, 10 sets of colon cancer samples with PES1 overexpression were analyzed, each set contains cancerous (T), adjacent (P), and distal (N) tissues from same patient. It was shown that 5 sets had high expression of c-Jun protein in the cancerous tissues (Fig. 5C). Additionally, an ORIGENE chip was used to detect the gene expression levels by real-time PCR. Quantitative analysis was performed using ΔΔCt method (2^−ΔΔCt^). As shown in Fig. 5D, tumors showed significant higher levels of PES1 and c-Jun expression in comparison with the normal colon tissues. Importantly, we observed a statistically significant co-expression of PES1 and c-Jun. c-Jun and PES1 gene expression have a correlation coefficient of r = 0.580 (P<0.0001) (Fig. 5E). These data suggested that there was a positive correlation between c-Jun and PES1 expression in colon cancer cells and tissues.

### JNK signaling pathway regulates PES1 promoter activity and expression

**Figure 6:**
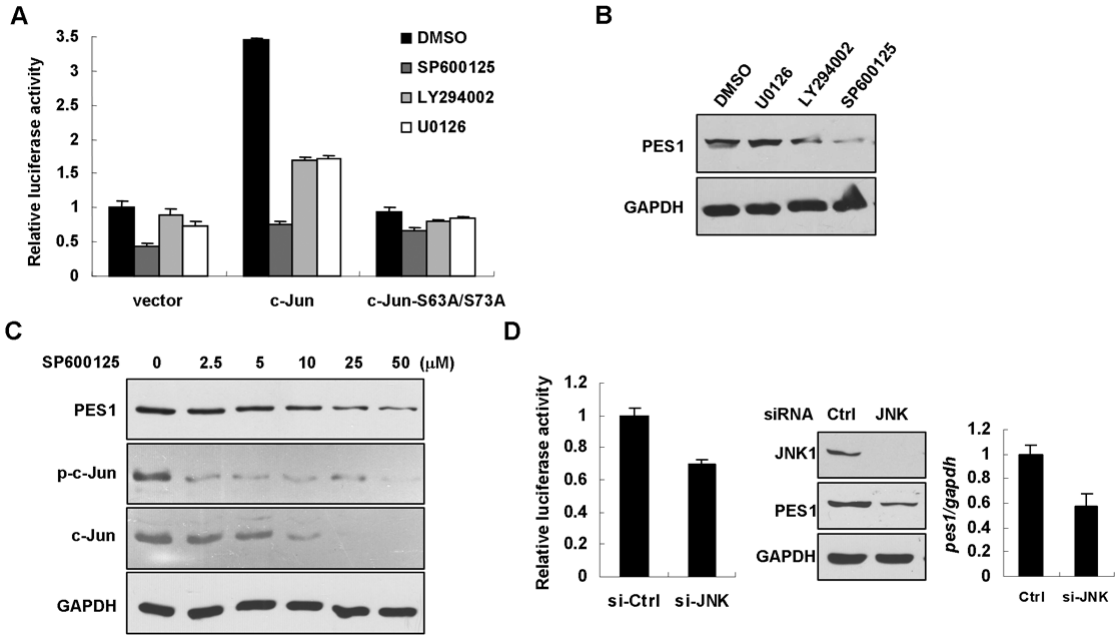
JNK signaling pathway regulates PES1 promoter activity and expression. (A) pGLB-PES11 (−274/+172) was co-transfected with c-Jun, c-Jun-S63A/S73A and control vector into HCT116 cells. After 48 hr of transfection, the cells were treated with the following inhibitors for another 36 hr, including U0126 (10 µM), LY294002 (10 µM), and SP600125 (10 µM). DMSO was used as control. Luciferase activity was normalized to Renilla luciferase activity.The luciferase of cells transfected with control vector, which was treated with DMSO, was set to 1. (B) Cells were treated with inhibitors as in (A) and Western blot analysis was performed to check PES1 expression regulated by these inhibitors in HCT116 cells. (C) Western blot analysis of PES1 expression affected by varying concentration of SP600125 (0–50 µM). Total and phosphorylated c-Jun were also examined. GAPDH was used as a loading control. (D) pGLB-PES1 (−274/+172) was co-transfected with siRNA-JNK or siRNA-control. After 72 hr of transfection, luciferase activity was detected and was normalized to Renilla luciferase activity. Expression of JNK1 and PES1 proteins were determined by Western blot. Expression of PES1 mRNA was detected by quantitative RT-PCR.

As a critical subunit of AP-1, c-Jun receives diverse upstream signals and hence, trans-activates downstream genes. To characterize signaling pathway(s) determining c-Jun-mediated transcriptional activation of PES1, HCT116 cells were transfected with pGLB-PES1 (−274/+172) containing the potential c-Jun binding site. After 48 hr of transfection, the cells were treated with a cohort of kinase inhibitors, including U0126 (MEK1/2 inhibitor), LY294002 (PI3 kinase inhibitor), and SP600125 (JNK inhibitor). Only treatment with SP600125 caused significant reduction in the luciferase activity (Fig. 6A). When the cells were co-transfected with pGLB-PES1 (−274/+172) plus c-Jun, SP600125 also exhibited strongest inhibition. Alternatively, in the presence of c-Jun-S63A/S73A, the effect of SP600125 was marginal (Fig. 6A). Based on these results, we proposed that JNK is the primary kinase maintaining the promoter activity of PES1. Consistent with this notion, Western blot analysis demonstrated that SP600125 profoundly inhibited PES1 protein expression in HCT116 cells, whereas U0126 and LY294002 had less effect (Fig. 6B). We further treated HCT116 cells with increasing amount of SP600125 and revealed that phosphorylation of c-Jun was gradually decreased, confirming that JNK activity of was inhibited. Correlated with theses alterations, PES1 expression was decreased by SP600125 in a concentration-dependent manner (Fig. 6C). Meanwhile, expression of c-Jun was lowered by SP600125, probably because phosphorylation of c-Jun is also required for maintaining its stability [27]. To better support the role of JNK in regulating PES1 expression, endogenous JNK1 was ablated by a small interference RNA (siRNA), in combination with transfection with pGLB-PES1 (−274/+172). It was shown that the promoter activity of PES1 was decreased by knock-down of JNK, in line with diminished mRNA and protein expressions of PES1 (Fig. 6D).

## Discussion

Mounting evidence has suggested that ribosomal proteins are frequently up-regulated in human cancer tissues [28], and the aberrant expression of the proteins regulateing rRNA processing is also associated with cancer and other human diseases [20], [29]. Our current study show that PES1 is overexpressed in colon cancers at both mRNA and protein levels, in comparison to the adjacent and normal colon tissues. Recently some other reports revealed PES1 overexpression in several other human cancers, including glioblastomas, breast cancer, and gastric cancer [1], [20], [22]. Kinoshita Y et al. found that the malignant human tumors exhibited up to 12-fold increase in PES1 expression relative to the adjacent tissues [1]. Protein microarray analysis revealed overexpression of PES1 in head and neck squamous cell carcinomas (HNSCCs), which was validated by Western blot and tissue microarrays containing 98 HNSCC specimens [19]. In an immunohistochemical analysis of breast cancer tissues, PES1 was detected in 88 of 92 (95.7%) cancer cases. And further quantitative analysis showed that the average mean optical densities (MODs) of PES1 staining in all stages of breast cancers were statistically higher than that in normal breast tissues [21]. Furthermore, PES1 was demonstrated to be one of the 79 genes upregulated in gastric cancer [20]. Our present result of PES1 overexpression in colon cancer tissues complements previous studies and further suggests that PES1 is upregulated in multiple human cancers, raising the possibility that PES1 may serve as a universal tumor marker and a potential therapeutic target, while the clinical significance of PES1 overexpression in colon cancer tissues and the prognostic value of PES1 needs be defined in future studies.

In previous studies, PES1 was reported to be essential for ribosome biogenesis, by which promoting cell proliferation [12], [13]. In this study, we demonstrated that silencing of PES1 inhibited the proliferation and colony formation of colon cancer cells. Xenograft graft growth was also impaired by PES1 silencing, suggesting that PES1 may function as an oncogene contributing to tumorigenesis. Through microarray screening, we further provided evidence that PES1 critically involved in determining expression of genes regulating cell proliferation. Interestingly, pathways related to cellular defense response, cellular biosynthetic process chemotaxis and transcriptional regulation, were also significantly affected by PES1 ablation, indicating PES1 is a multifunctional protein playing potential roles in distinct biological processes. How PES1 regulates expression of such many genes is unclear, but a previous study reported that PES1 may modulate gene expression by directly binding to certain DNA sequence [15]. The mechanism underlying PES1's diverse functions is under investigation.

Although PES1 was found to be overexpressed in multiple human tumors, little was known about the mechanism controlling PES1 expression. The human PES1 gene localizes at chromosome 22q12.1, a locus has not been reported to be amplified. Recently, through somatic quantitative multiplex PCR for short fluorescent fragments (QMPSF) screening of 56 colon cancer tissues, only 5.4% (3/56) was identified to harbor increased copy number of PES1 gene [30]. Thus, increased expression of PES1 protein in colon cancers can not be fully explained by gene amplification. Herein, for the first time, we characterized the role of c-Jun in transcriptionally activating PES1 expression. AP-1 is a sequence-specific transcriptional factor composed of Fos and Jun family members, which form homo- or heterodimers to recognize the AP-1 site or related sequence. As one of major subunits of AP-1 complex, c-Jun was reported to upregulated in some human cancers [31]. We found that c-Jun significantly increased the PES1 promoter activity in luciferase assay, but JunB, JunD, and c-Fos had less stimulatory effect. Furthermore, c-Fos abolished c-Jun-induced activation of PES1 promoter in the co-transfection assay, implying that c-Fos is a negative regulator antagonizing the effect of c-Jun in cells utilized.

AP-1 is often the final target of intracellular kinase signaling cascades and is activated to regulate the expression of downstream genes by binding to the promoter [32]. Our data revealed that transcriptional activation of PES1 by c-Jun is dependent on JNK in colon cancer cells. In human cancers, several studies have reported that “hyperactive” signaling pathway resulting from elevated JNK activity. For example, JNK activity is increased by 23 fold in colonic neoplasms [33]. Moreover, in some recent studies it was demonstrated that suppression of JNK activity decreased tumor metastasis [34], [35]. As a cognate substrate of JNK, serine63 and serine73 of c-Jun are phosphorylated by JNK, modifications required for maintaining c-Jun's transcriptional activity and its stability [26], [27]. By using mutant c-Jun defective for phosphorylation, and chemical inhibitor SP600125, we also demonstrated an essential role of JNK-mediated phosphorylation of c-Jun in controlling PES1 expression, suggesting that suppression the expression of PES1 by blockade of JNK activity could be of therapeutic potential for cancer treatment.

## Supporting Information

Figure S1 **Generation and characterization of PES1 monoclonal antibodies.** (A) ELISA analysis of specificity of PES1 mAbs. Supernatants from clones of hybridoma cells were incubated with GST and GST-PES1 in a 96-well plate, followed by detecting with HRP-conjugated anti-mouse IgG antibody and then with substrate. Anti-GST mAb and normal mouse IgG were used as a positive and a negative control, respectively. (B) Western blot analysis for specificity of PES1 mAbs using GST-PES1 and GST protein (10 ng). Anti-GST mAb and normal mouse IgG were used as a positive and a negative control, respectively. (C) Western blot analysis of PES1 mAbs with endogenous PES1. Cell lysates containing 50 µg of total protein from AGS cells were detected with indicated anti-PES1 mAbs. The normal mouse IgG were used as a negative control, the blots were probed with anti-β-Actin to ensure equal loading. (D) Western blot analysis of RNA-interference of PES1. AGS cells were transiently transfection of a shRNA targeting PES1 mRNA or an unrelated shRNA as a negative control. After 48 hr, cell lysates from AGS cells were processed for Western blot with anti-PES1 mAb 3B1. (E) ELISA analysis for comparison of PES1 mAbs and a commercialized polyclonal antibody (Bethyl Laboratories). Purified anti-PES1 mAb 3B1 and commercial antibody were incubated with protein of GST and GST-PES1 in a 96-well plate, followed detecting with HRP-conjugated anti-mouse IgG antibody and then with substrate. (F) Western blot analysis for comparison of PES1 mAbs and commercial antibody. Cell lysates containing 50 µg of total protein from AGS cells were processed for Western blot with indicated purified anti-PES1 mAb 3B1 and commercial antibody at the same concentration 0.2 µg/ml. The normal mouse IgG were used as a negative control, and the blots were probed with anti-β-Actin to ensure equal loading. (G) Immunocytochemistry analysis for comparison of PES1 mAbs and commercial antibody. AGS cells grown on coverslips to 50% confluence were fixed with 4% paraformaldehyde in PBS and incubated with purified anti-PES1 mAb 3B1 and commercial antibody at the same concentration 1 µg/ml, normal mouse IgG was used as a negative control. Then coverslips were then incubated with the peroxidase based EnVisionTM kit. The results were observed microscopically.
(TIF)

Figure S2 **Microarray analysis with PES1 silenced HCT116 cells.** (A) Pathways affected by PES1 ablation with Gene Ontology Analysis tool. These pathways were ranked according to the significance (P value). (B) Validation of some of the down-regulated and up-regulated genes' expression by quantitative RT-PCR in HCT116 and SW480 cells. *Gapdh* was used as a housekeeping gene.
(TIF)

Table S1 Primers of quantitative RT-PCR for validating the results of Microarray analysis.
(DOC)
