# Supplementary figures and images for: Transcriptional Regulation of PES1 Expression by c-Jun in Colon Cancer
Source: PLoS One. 2012 Jul 30;7(7):e42253. doi: 10.1371/journal.pone.0042253 (PMC3408486; doi:10.1371/journal.pone.0042253)

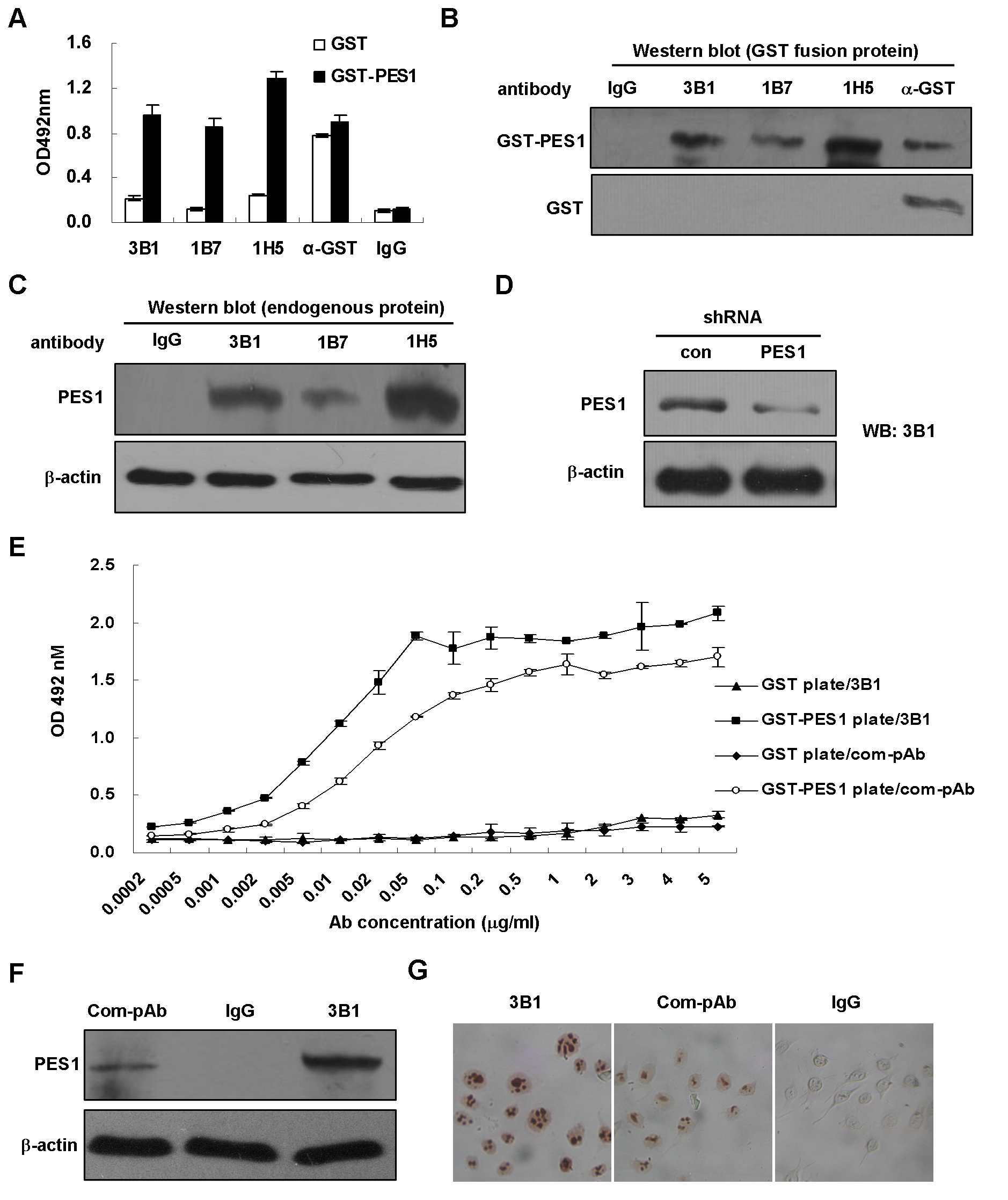

Supplement: Figure S1 — Generation and characterization of PES1 monoclonal antibodies. (A) ELISA analysis of specificity of PES1 mAbs. Supernatants from clones of hybridoma cells were incubated with GST and GST-PES1 in a 96-well plate, followed by detecting with HRP-conjugated anti-mouse IgG antibody and then with substrate. Anti-GST mAb and normal mouse IgG were used as a positive and a negative control, respectively. (B) Western blot analysis for specificity of PES1 mAbs using GST-PES1 and GST protein (10 ng). Anti-GST mAb and normal mouse IgG were used as a positive and a negative control, respectively. (C) Western blot analysis of PES1 mAbs with endogenous PES1. Cell lysates containing 50 µg of total protein from AGS cells were detected with indicated anti-PES1 mAbs. The normal mouse IgG were used as a negative control, the blots were probed with anti-β-Actin to ensure equal loading. (D) Western blot analysis of RNA-interference of PES1. AGS cells were transiently transfection of a shRNA targeting PES1 mRNA or an unrelated shRNA as a negative control. After 48 hr, cell lysates from AGS cells were processed for Western blot with anti-PES1 mAb 3B1. (E) ELISA analysis for comparison of PES1 mAbs and a commercialized polyclonal antibody (Bethyl Laboratories). Purified anti-PES1 mAb 3B1 and commercial antibody were incubated with protein of GST and GST-PES1 in a 96-well plate, followed detecting with HRP-conjugated anti-mouse IgG antibody and then with substrate. (F) Western blot analysis for comparison of PES1 mAbs and commercial antibody. Cell lysates containing 50 µg of total protein from AGS cells were processed for Western blot with indicated purified anti-PES1 mAb 3B1 and commercial antibody at the same concentration 0.2 µg/ml. The normal mouse IgG were used as a negative control, and the blots were probed with anti-β-Actin to ensure equal loading. (G) Immunocytochemistry analysis for comparison of PES1 mAbs and commercial antibody. AGS cells grown on coverslips to 50% confluence were [file pone.0042253.s001.tif]

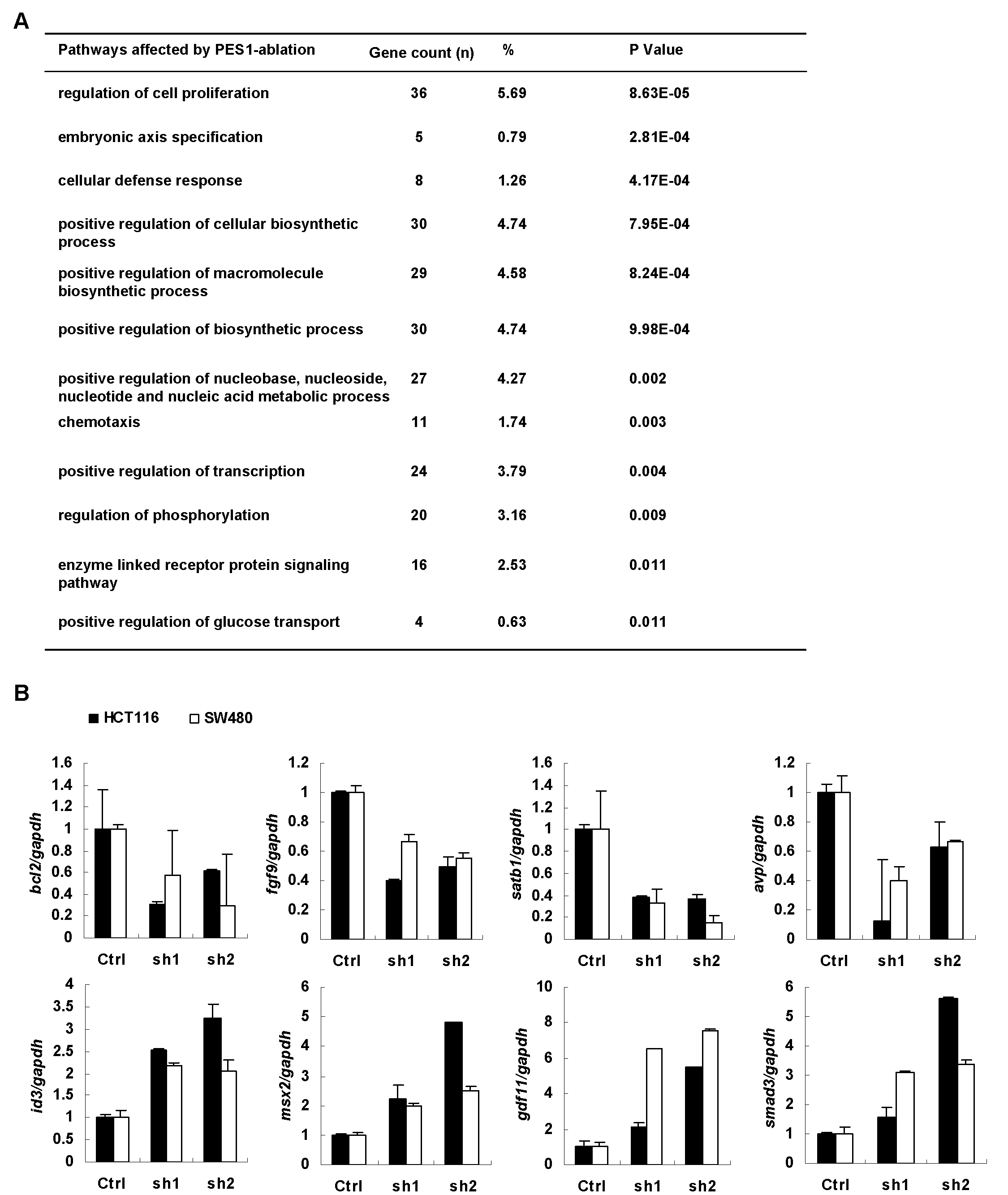

Supplement: Figure S2 — Microarray analysis with PES1 silenced HCT116 cells. (A) Pathways affected by PES1 ablation with Gene Ontology Analysis tool. These pathways were ranked according to the significance (P value). (B) Validation of some of the down-regulated and up-regulated genes' expression by quantitative RT-PCR in HCT116 and SW480 cells. Gapdh was used as a housekeeping gene. (TIF) [file pone.0042253.s002.tif]
