# Supplementary material for: Transcriptional Regulation of PES1 Expression by c-Jun in Colon Cancer
Source: PLoS One. 2012 Jul 30;7(7):e42253. doi: 10.1371/journal.pone.0042253 (PMC3408486; doi:10.1371/journal.pone.0042253)
Supplement: Table S1 — Primers of quantitative RT-PCR for validating the results of Microarray analysis. (DOC) [file pone.0042253.s003.doc]

Supplementary Table1. Primers of quantitative RT-PCR for validating the results of Microarray analysis.

| Gene | Forward primer | Reverse primer |
| --- | --- | --- |
| *AHR* | AGTCTCCCTTCATACCTT | TTGCATGTGCTTCATCTTCT |
| *BCL2* | TGCACCTGACGCCCTTCACC | GGGCGACATCTCCCGGTTGA |
| *ID3* | GCGGTGTGCTGCCTGTCG | ACCTGGCTAAGCTGAGTG |
| *SMAD3* | AGTCTCCCAACTGTAACCA | AAACTCCTGGTTGTTGAAGA |
| *GDF11* | CGGGAGGTAGTGAAGCAG | GGAAATGACAGCAGAGAGG |
| *AVP* | TGTGTGCACCAGGATGCCT | TTCTGGAAGTAGCACGCGG |
| *FGF9* | AATGTGCCCGTGTTGCCGGT | CCAAGTCCGTGACTGCGGGT |
| *SATB1* | CATTCAAGCTCCTTTCCCTTTC | TGGGCTCGTATCAACACCTATC |
